# Post-Traumatic Stress Disorder After Acute Cardiovascular Events: Protocol of a Systematic Review and Meta-Analysis

**DOI:** 10.3390/jcm15082962

**Published:** 2026-04-14

**Authors:** Harleen K. Sandhu, Michael P. Van Wie, Mary B. Short, Charles C. Miller

**Affiliations:** 1 Department of Cardiothoracic and Vascular Surgery, McGovern Medical School at UTHealth Houston, 6400 Fannin St, Ste. #2850, Houston, TX 77030, USA; charles.c.miller@uth.tmc.edu; 2 Department of Psychological and Behavioral Health Sciences, University of Houston-Clear Lake, Houston, TX 77058, USA; vanwie@uhcl.edu (M.P.V.W.); shortmb@uhcl.edu (M.B.S.)

**Keywords:** post-traumatic stress disorder, acute cardiovascular events, aortic disease, medical trauma, PTSD

## Abstract

**Background:** An aortic disease diagnosis can be perceived by patients as a stressful and often life-altering event. In addition, an acute event, such as aortic rupture or dissection—and the surgical intervention that will be required to address it—can be viewed as potentially life-threatening and traumatic. Serious health conditions, including stroke and acute coronary syndromes, have been described in the literature to correlate with trauma-like symptoms. Post-traumatic stress disorder (PTSD) is well described in connection with external traumatic events, such as war, assault and similarly catastrophic events. A key element of this type of PTSD is that its occurrence arises secondary to an external traumatizer. However, recent work has suggested that internal events—such as a catastrophic medical event (e.g., acute cardiovascular event and/or surgery)—can trigger PTSD symptoms. An important question is whether medical event-initiated PTSD can (or should) be treated similarly to traditionally defined PTSD, when the triggering threat may persist rather than having been confined to a past event. This systematic review will summarize the literature on the occurrence of PTSD as a consequence of an acute cardiovascular event and attempt to identify effective treatments using meta-analysis, if the literature quantity and quality support it. **Methods:** The search strategy will include publicly available electronic databases, including MEDLINE via PubMed and OVID, EMBASE via Elsevier, Cumulative Index for Nursing and Allied Health Literature (CINAHL) via EBSCOhost, SCOPUS, PsychInfo, and the Cochrane Library, to identify publications that report the development and/or treatment of PTSD as a consequence of an acute cardiovascular event, which include cardiac arrest, acute coronary syndromes, and acute aortic syndromes. Identification of publications, article classification, methodological review/quality assessment, and data extraction will be performed by two trained experts in cardiovascular epidemiology, with the resolution of disagreements carried out by a third independent reviewer. The review conduct and meta-analysis will follow PRISMA and MOOSE guidelines. Data will be aggregated using random effects models when quantitative data are reliable and heterogeneity is reasonable. If a quantitative synthesis is not possible due to data quality, a narrative synthesis will be conducted. Statistical heterogeneity will be assessed by I^2^ statistics. The quality of evidence will be assessed using the GRADE criteria. **Ethics and Dissemination:** This study did not require an institutional review board or human subjects protection committee approval given the nature of the study design. The results will be published in a peer-reviewed journal, along with recommendations for future research.

## 1. Introduction

Despite advances in medical and surgical interventions, cardiovascular disease (CVD) remains the global leading cause of morbidity and mortality [1]. Since the early 1990s, chronic stress disorders, especially post-traumatic stress disorder (PTSD), have been linked to the development of CVD [2]. Research has established that PTSD has a complex, bidirectional interplay with incident CVD, which means PTSD can be both a consequence and a cause of CVD [3,4]. Furthermore, PTSD has been linked to specific types of events that relate to CVD. The development of PTSD as a consequence of CVD has been described more commonly in the context of acute cardiac events, such as acute coronary syndromes (ACSs), including myocardial infarction (MI), unstable angina (UA), and sudden cardiac arrest (CA) [5,6]. More recently, attention has been drawn toward acute aortic syndromes (AASs) and their traumatogenic potential [7,8]. However, significant knowledge gaps persist in the current understanding of the relationship between such acute CVD events and the development of PTSD. Furthermore, the treatments that could be effective for this specific condition (PTSD secondary to acute CVD) are poorly understood.

Thus, the following sections will first summarize what is known about the bidirectional association between CVD and PTSD, highlighting the gaps in understanding, especially related to PTSD as a consequence of acute CVD. Then, specific CVD events, CA, ACSs and AASs, will be described in detail, which will include the current gaps in the literature related to the link between PTSD and acute CVD events. Last, the limited research on the treatments specific to CVD-linked PTSD will be reviewed.

### 1.1. Current Research on PTSD-CVD Link

Given that CVDs may be associated with PTSD, it is important to understand what is known. Overall, PTSD has been shown to be comorbid with, and likely an independent risk factor for, CVD, with a 53% increase in incident CVD identified in patients with PTSD symptoms compared to those without (with 27% persisting after adjustment for comorbidities) in a meta-analysis of five prospective studies [9]. INTERHEART, a case–control study conducted with 30,000 participants in 52 countries, found that stressful life events accounted for about one-third of the population attributable risk (PAR) for MI, with a PAR estimate of 32.5% [10]. Atrial fibrillation, a form of cardiac rhythm abnormality affecting the upper chambers (atria) of the heart, has also markedly increased in patients with PTSD symptoms, both occurring at a higher rate and occurring earlier in life than in patients without PTSD symptoms, with a reported hazard ratio of 1.3 (1.1 after adjustment) [11]. Furthermore, studies have shown that positive PTSD screens are common in patients with CVD, occurring in 12% of patients with ACS and 23% with stroke or CVD [9].

Autonomic dysregulation is the most commonly described underlying mechanism linking PTSD and CVD [3,4,9,12]. Heart rate and blood pressure increase with PTSD as a result of increased sympathetic (fight or flight) nervous system activation [13,14]. In normal circumstances, the parasympathetic/vagal response “turns down” the heightened arousal resulting from sympathetic activation in a process termed “allostasis.” When allostasis becomes dysregulated and sympathetic arousal is prolonged over time—referred to as “allostatic load”—this can lead to numerous long-term effects in the body, particularly in the circulatory system [14]. A specific hazardous condition for the development of coronary disease is dysfunction of the vascular endothelium. This is the cellular layer that protects the insides of arteries from injury and keeps them open (patent) with blood flowing effectively [15]. When the endothelium does not function normally, vascular spasms can occur, causing a temporary restriction of blood flow, along with a damaged endothelial lining. This can result in platelet aggregation at the injury site with formation of atherosclerotic plaques [15]. These plaques can slowly block arteries over time and, in critical locations, rupture. Rupture often results from a catecholamine surge caused by a stressful event. In addition, they can result in immediate injury to critical cardiovascular structures, including the heart and the aorta [16]. Endothelial dysfunction and the cascade of maladaptive responses that result from it are potentiated by a chronic pro-inflammatory state [9], which is a signature element of PTSD [17]. Behavioral factors secondary to PTSD have also been implicated in the interplay between PTSD and CVD, including smoking and substance use, physical inactivity and medication non-adherence [9]. Medical/pharmacologic burden-reduction strategies—such as combining multiple medications into a single “polypill”—have been described as improving patient medication adherence among CVD patients [18], though the effect of this strategy on the PTSD-CVD link remains unknown.

As mentioned, there exists a bidirectional relationship between CVD and PTSD [3,4], where PTSD has been implicated as both a cause (as described thus far) and a consequence of CVD, particularly after an acute CVD event. Historically, most of the literature on the link between PTSD and CVD has been focused on PTSD as a cause of CVD. However, new evidence has been emerging that PTSD is not only a cause but also a consequence of traumatic cardiovascular events, with events, such as MI or CA, identified that can initiate PTSD onset [9,12,14,19,20,21,22]. Additionally, medical interventions, such as open-heart surgery or implantable cardioverter defibrillator (ICD) implantation, can also be traumatogenic [23]. ICDs have specifically been studied in relation to PTSD because they are inherently distressing medical devices. The shocks delivered by ICDs are unpredictable, seem to the patient to occur randomly, and are painful and extremely distressing. ICDs have been associated with the development of PTSD [24], both because of the pain and suddenness of being shocked and the startling report of a potentially fatal event unfolding in real time [25].

An estimated one in eight survivors of ACS go on to develop PTSD [3]. Once established, this acute CVD-induced PTSD may increase the risk of subsequent CVD events, including cardiac death, by as much as two-fold [3], especially after CA, which is particularly traumatic [26]. Evidence suggests that PTSD symptoms, especially intrusive symptoms, as a result of an acute CVD event, are significantly associated with poor long-term outcomes, such as major adverse cardiac events and all-cause mortality [27].

Acute CVD events are likely to engender consequent PTSD through a variety of mechanisms. Part of the problem is heightened autonomic arousal, which increases heart rate, blood pressure and vascular tone and, over time, affects metabolic processes that lead to derangements, such as type 2 diabetes and metabolic syndrome [28]. As accumulated stress becomes established post-event and allostatic load becomes persistently increased, the neurohumoral effects that arise from this stress affect vascular endothelial function similarly to conventionally defined mechanisms of PTSD [14,16]. Other biopsychosocial and behavioral manifestations may also include poor sleep quality and medication adherence problems as a consequence of the avoidance component of PTSD [9,12,21,29].

One issue with the current literature is how CVD events are defined. A lot has been written about cardiovascular events—particularly acute coronary events—as they relate to PTSD. However, little has appeared in the literature involving AAS events. In fact, when describing PTSD as a consequence of an acute CVD event, most studies report CVD events, such as ACS and, less often, CA. However, AASs that constitute another important life-threatening acute CVD phenomena are often not included in these definitions or reports. Recently, the Aortic Dissection Collaborative has identified important decrements in health-related quality of life among survivors of acute aortic dissection, a type of acute CVD, and surveys have identified the aortic-dissection event as “a turning point” and an existential threat to life and autonomous living [7]. Therefore, a clearer and more inclusive definition of acute cardiovascular events (ACEs) is needed, and the literature associated with ACEs and PTSD is in need of a thorough review.

### 1.2. Acute Cardiovascular Events

#### Defining ACEs

ACEs, as defined herein, include CA, ACSs—which include MI of the ST-elevation (STEMI) and non-ST-elevation (NSTEMI) varieties—UA [30,31] and AASs—which include acute aortic dissection, penetrating aortic ulcer, and intramural hematoma [32]. CA is an event in which the heart stops beating effectively and cannot pump blood due to some sort of heart rhythm disturbance (arrhythmia), which may be caused by direct injury to the heart muscle (myocardium). CA can result from abnormal electrical activity in the heart, or from damage to the heart’s structure and electrical conduction apparatus, due to coronary perfusion defects, resulting in myocardial injury and tissue destruction [33].

ACSs more explicitly involve direct injury to the myocardium with permanent tissue destruction and include STEMI, NSTEMI and UA [31]. With each normal heartbeat, an electrical impulse, as captured in the electrocardiogram (ECG), propagates in waves of activity, described as P, Q, R, S and T waves [34]. The P wave starts the sequence, where the atrial chambers undergo electrical depolarization and contract, ejecting blood from those chambers. Next, the QRS complex occurs, which represents depolarization and contraction of the left and right ventricles. The ventricles are the lower chambers of the heart that eject blood to the lungs on the right side and the arterial circulation on the left. The QRS complex is followed by the T wave, in which the ventricles repolarize and prepare to respond to the next cardiac cycle impulse [34]. The time between the S and T waves is known as the “ST interval” or “ST segment.” This segment is particularly important for understanding the state of the myocardium. The time course and the height of the ST segment relative to the baseline of the Q wave on the ECG gridline contain information about how the myocardium is conducting the constituent signals [34]. When the ST segment is elevated from the baseline, this indicates a serious abnormality in which the myocardium suffers a through-and-through transmural injury [30]. This type of injury can be immediately fatal, and requires immediate revascularization, using thrombolytic (clot-dissolving) medications and cardiac catheterization to restore blood flow as soon as possible [31]. Transmural myocardial infarcts are caused by total or near-total occlusion of major coronary arteries. Such occlusions usually result from ruptured atherosclerotic plaques [35]. Plaques in turn result from many of the previously mentioned risk factors related to PTSD, such as chronic inflammation, lipid and metabolic dysregulation, stress hormones, endothelial dysfunction, and related behavioral and psychosocial factors, including smoking, physical activity, age and poor nutrition [15].

Non-ST segment elevation MI (NSTEMI) ECG findings represent less severe types of infarcts than STEMI, which are confined to the wall (subendocardium) or middle (intramural) portion of the muscle but do not extend transmurally through the full thickness of the muscle. NSTEMIs remain serious and can cause lasting damage, and may result in the need for additional stressful interventions, such as a need for ICDs, which deliver shocks to the heart muscle when abnormal rhythm is detected [35]. In extensive clinical trial experience, ICDs have been shown to reduce mortality [36,37] and affect patient quality of life in ways that are not always positive [38]. That is, even though NSTEMI is not as acutely life-threatening as STEMI, medical countermeasures used to manage injuries from NSTEMI can be trauma-inducing and result in the development of PTSD [24].

UA is often a prodrome to an MI or CA, meaning that angina is a grave sign; however, STEMI and NSTEMI can happen without the warning of chest pain or other typical symptoms [39]. Although each of these ACSs (UA, STEMI, NSTEMI) are physiologically distinct entities, the patient’s experience of these traumatic events can result in a similar psychological stress response and the development of PTSD in its aftermath.

A type of ACE that has not been studied extensively in relation to PTSD is AAS, which we argue should be added to the definition of ACE. AAS is defined as acute dissection (AD), acute intramural hematoma (IMH) and penetrating aortic ulcer (PAU) [32]. AD, especially type-A AD, which affects the ascending aorta and aortic arch, is a vascular catastrophe that is immediately life-threatening, with historical mortality rates of roughly 1% per hour for the first 48 h after onset [40]. IMH and PAU are considered AASs because they are likely unstable and can result in acute-phase mortality [41]. All AASs involve defects in the aortic wall, which may lead to life-threatening leaks, expansion or rupture [32]. The aorta carries the entire arterial blood volume out of the heart and is an enormously consequential place to have a vascular rupture. Patients with AAS are younger and generally less ill than patients with ACS and, unlike coronary or valvular surgery—where part of the benefit of intervention is immediate improvement in physically limiting symptoms—major aortic surgery is performed primarily to repair a defect in the aorta and does not result in symptomatic improvement [7]. Given this lack of symptomatic improvement with such major surgery, aortic surgery may be perceived as relatively more burdensome than coronary surgery or catheter-based intervention [7]. Additionally, treatment compliance is particularly complex and challenging for patients after an aortic catastrophe and aortic surgical repair. This is because good management requires a high level of medication compliance, strict adherence to imaging and clinic visit follow-up, and a fairly high level of health literacy to understand how risk factor control is related to recurrence risk [42,43].

ACEs are inherently life-threatening and patients who survive them often worry about the event happening again—and may become hypervigilant regarding the monitoring of cardiovascular symptoms. In fact, hypervigilance toward bodily sensations as a result of fear of recurrence has been described as an ongoing challenge for AAS patients—and this may continue to feed health anxiety and prolong the trauma experience [8]. Thus, AASs are of particular concern because, although much more rare than ACS events but similar to ACSs and CA, they are life-threatening and have a very high near-term mortality and typically require immediate open-heart surgery. They also carry a significantly elevated long-term risk [32]. Given these consequences, it seems logical that PTSD is often associated with an ACE.

### 1.3. PTSD After an ACE

Another important area that needs further understanding is PTSD being a result of CVD or ACEs. Overall, although the existing literature in this topic area has thoroughly examined CVD as a consequence of PTSD, research related to PTSD occurring after an ACE is still in its infancy. The literature regarding PTSD as an outcome of ACEs requires review and a synthesis of the research.

Overall, PTSD is defined by the Diagnostic and Statistical Manual of Mental Disorders Criterion (5th ed.; DSM-5) [44] as a trauma/stress-related disorder resulting as a consequence of a traumatic event (Criterion A) and is characterized by the following symptoms: intrusions or re-experiencing the trauma (Criterion B); avoidance (Criterion C); negative alterations in cognition and mood (Criterion D); and arousal/reactivity (Criterion E).

Since 1980, Criterion A has been a foundational element of the DSM definition of PTSD and has also been the most debated across multiple versions of the DSM (DSM III-5), due in particular to controversy about what kinds of events are classified as “traumatizers” vs. “stressors” [45,46].

Beyond the headline criteria of the event as threatening life or bodily integrity, the text of the DSM IV indicated for the first time that “being diagnosed with a life-threatening illness” could qualify as a Criterion A event [47]. In this framework, ACEs would clearly qualify. However, with DSM-5’s iteration of “Criterion A,” a contrary perspective began to emerge with the introduction of the cautionary language stating “a life-threatening illness or debilitating medical condition is not necessarily considered a traumatic event” [44]. This introduces uncertainty into which types of illnesses would qualify as “stressors” and which as “traumatizers.” This distinction becomes important because the DSM-5 recommends diagnosing stress-inducing events that occur in the absence of Criterion A events as adjustment disorders (DSM-5) [44] rather than as PTSD. The ambiguity of the new classification casts doubt on whether ACE-related PTSD can be classified as PTSD at all. This may have important implications for treatment.

ACEs are life-threatening, sudden events. The unpredictability of these acute events and the imminent risk of fatality—along with the patient’s perceived loss of control and ensuing helplessness during/after the event—contribute to the unique traumatizing character of the patient experience [22,48]. There is a growing body of evidence that such cardiovascular event-induced traumatic experiences, and ensuing medical or surgical treatments, can result in significant emotional distress and in post-traumatic stress symptoms or full-blown PTSD [22,23,48,49]. This accumulated evidence supports the case that ACEs, which are injuries that violate bodily integrity, are sufficient to serve as traumatic events that fall under criterion A of DSM-5 for diagnosis of PTSD [44], despite the new cautionary language about medical events “not necessarily” being traumatic.

### 1.4. ACEI-PTSD Extends the Traditional Conceptualization of PTSD

For our purposes, PTSD arising from an ACE (as defined above) is termed as ACE-induced PTSD or ACEI-PTSD. PTSD is described as being among the Trauma and Stress Disorders in the DSM-5 (309.81) [44]. A hallmark characteristic of traditionally defined PTSD is intrusiveness of thoughts and flashbacks to a past traumatic event (combat, rape, natural disaster, etc.), which continues to distress the affected person despite the actual threat no longer being present [21].

ACEI-PTSD, like traditionally defined PTSD, arises initially from a past life-threatening traumatic event. However, in distinction from traditional PTSD, it focuses on realistic considerations of recurrence that other forms of trauma-related disorders do not. That is, ACEI-PTSD involves not only flashbacks of the primary traumatic event (ACE) but this intense fear of ongoing progression/recurrence of disease or flashforward cognitions to probable future events, and may involve debilitating perseverations about potential future events, such that the underlying fear of mortality sustains persistent PTSD symptoms [48,50].

### 1.5. Enduring Somatic Threat Model

In 2014, Edmondson authored the Enduring Somatic Threat (EST) model of medical trauma-induced PTSD as a way to conceptualize the unique features of this phenomenon and to distinguish it from the traditional understanding of PTSD [50]. This work represents a “Copernican turn” in the field of PTSD research—conceptually resonant with Copernicus’ 1543 realization that the earth orbits the sun and not vice versa. Similarly, while traditional PTSD relates to emotional distress and symptoms resulting from triggers of past events (for which recurrence is unlikely), medical trauma-induced PTSD is subject to a meaningfully ongoing risk recurrence. That is, a persistent EST remains for medical trauma that differentiates it from more traditionally understood triggers of PTSD [50]. This further underscores the difference in the very nature of the source of threat or trigger. While the traditional understanding of “threat” in connection with a traumatic event arises from an external source like a disaster, accident, assault, etc., the EST model posits that the threat in medical trauma-induced PTSD lies internal to the patient, such as recurrent CVD-related events, disease progression, implantable devices, etc. The “threat” is very real in that the chances of a patient having another cardiac event or needing a surgical procedure or experiencing worsening of disease are considerably higher after an initial event. Thus, there is a component of “*flashforward cognitions*” [21,49,50] added to the traditional definition of intrusive symptoms of PTSD (Criterion B for PTSD in DSM-5) [44].

Differences in avoidance behavior are also important elements of distinction between these two PTSD perspectives. In traditionally understood PTSD, avoidance behavior can be pathological, in that patients can avoid exposure to external triggering stimuli to the extent that it interferes with mood and social functioning and may worsen social isolation and depressive symptoms [29]. EST, in contrast to the usual PTSD characterization, cannot be avoided—it exists within the body and the potential for event recurrence the patient is exposed to is probable. The subject of intrusive threat cognitions is not avoidable. Rather, avoidance tends to transfer to health behaviors, such as medication adherence and other health-impacting activities [50].

Furthermore, patients with ACEI-PTSD might experience physical symptoms, such as elevated heart rate or palpitations, which can be perceived as a sign portending cardiac catastrophe, thereby causing worsening of mood/affective and hyperarousal symptoms (Criterion D and E for PTSD in DSM-5) [44]. This differs from traditional PTSD, where such physical symptoms are experienced in the context of reminders of the past traumatic event. This potential for misinterpreting physical symptoms and difficulty in distinguishing their CVD-related vs. trauma-related cause among ACEI-PTSD patients further highlights the uniqueness of medical trauma-induced PTSD from traditional trauma disorders as well as the need for more specialized interventions [21,51].

ACEI-PTSD, with its EST, may require an expanded approach for treatment beyond what has traditionally been described for PTSD. As mentioned, as PTSD symptoms might manifest differently in ACEI-PTSD, such as avoidance behaviors resulting in poor medical treatment compliance or misinterpretation of physical symptoms (CVD vs. trauma-related), this may require focused therapeutic interventions, such as ACE-informed psychoeducation. The complex medical and sociocultural milieu involving an ACE, like AAS, may require extended psychological intervention planning for the patient and their immediate support network [7]. The description so far has hit the highlights in the literature that call out these types of concerns and, although interest is gathering, the literature regarding trauma-focused approaches to handling PTSD in this setting is lacking and needs addressing.

### 1.6. Treatment Interventions: Effects of PTSD Variants on Treatment Effectiveness

In treatment studies of psychological interventions for PTSD of traditional origin, trials demonstrate that cognitive behavioral therapy (CBT) is the gold standard [14]. Other specific behavioral interventions (e.g., exercise) can reduce inflammatory biomarkers and other indicators of maladaptive psychophysiological response [4,12]. Social support is also a critical element that affects resilience to PTSD, but the perceived positivity of such support is crucial to its effectiveness as a buffer against the stress response. Social interactions may have an inverse association with symptomatic improvement if those interactions are perceived to be negative or lacking understanding [52]. The influence of quality of social support may call for emotionally focused therapy (EFT), involving multiple members of the social support network in circumstances where key social contacts (caregivers, family members, partners) are not fully engaged and supportive [14].

Evidence-based interventions, such as CBT and exposure therapy, have been shown to be effective in PTSD, with trauma-focused therapies providing superior reductions in symptom severity relative to non-trauma-focused therapies [21]. Less is known about how well these findings extend to ACEI-PTSD. For instance, not many studies have focused on the ACEI variant of PTSD but one showed that EMDR was more effective in reducing symptoms after cardiac surgery than CBT [53], which is not necessarily the case in traditionally defined PTSD. Another study comparing imaginal exposure therapy to psychoeducation showed neither differences between treatment groups nor improvement in symptoms following treatment in the general population. However, it showed some improvement in the most severely affected patients who had recurrent CVD events and high starting symptom scores [54].

Given the ongoing and forward-focus of ACEI-PTSD, the results from the standard variant PTSD literature may not generalize, and further research is needed. An additional challenge for managing ACEI-PTSD is that many of the assessment instruments currently used for PTSD do not capture all of the defining clinical features of this variant—and much of the prior work is based on instruments still modeled on DSM IV, which does not use the current DSM 5 criteria for PTSD as a chronic stress disorder [21].

### 1.7. The Study’s Rationale and Goals

The preceding background provides a summary of important concepts that have described links between CVD, including ACSs, with PTSD, especially with regard to their bidirectional relationship. However, as highlighted in the previous sections, gaps remain in the understanding of the link of PTSD consequent to ACEs and the treatments that may be most effective for ACEI-PTSD. More specifically, some important knowledge gaps that need further consideration include how previously described ACE events have not included AASs, whether traditional PTSD psychological/behavioral interventions are appropriate for ACEI-PTSD, the effectiveness of such interventions on PTSD- and ACE-related outcomes, and whether existing trauma-focused therapies affect CVD health-harming behaviors and clinical outcomes.

This review’s primary goals are twofold. First, to comprehensively summarize the literature and provide a thorough understanding of ACEI-PTSD and existing gaps in knowledge. (To the best of our knowledge, this will be first formal systematic review of its kind that will synthesize the existing literature to describe the prevalence and characterization of PTSD symptoms arising as a consequence of ACEs [using the expanded definition to include AASs] and across all ACE subtypes.) Second, to support a meta-analysis to evaluate the effectiveness of psychological interventions (trauma-focused interventions vs. non-trauma-focused interventions vs. other non-EBI or no interventions) on clinical (mental health as well as CVD-related) outcomes in patients with acute cardiovascular event-induced stress disorder or ACEI-PTSD.

An important objective is that the results of the review and meta-analysis will provide a comprehensive body of evidence that can be used to set a research agenda. The review will assess the quality of recommendations in the current literature based on the level of evidence supporting them and propose topics for therapeutic trials to help answer important questions about which strategies are likely most promising. Finally, using the information in this review, details related to guidelines and policy for implementing and promoting an integrated approach (comprehensive care model involving mental and physical health) toward the management of patients with ACEI-PTSD will be addressed.

### 1.8. Specific Aims

1. To summarize the existing literature on the prevalence and characterization or presentation of ACEI-PTSD, including CA, ACSs, and AASs;
2. To summarize the treatments being used specifically for ACEI-PTSD, in the subset of articles addressing interventions, and categorize them (e.g., trauma-focused vs. non-trauma-focused EBI vs. non-EBI) and assess their effectiveness for both PTSD symptom reduction and hard clinical outcomes (recurrent events, death);
3. To assess the quality of evidence to determine the feasibility of developing a framework for preliminary practice guidelines for ACEI-PTSD based on the current state of the science. A practice guideline framework would include strength-of-recommendation guidance based on levels of existing evidence.

## 2. Methods

The protocol for this systematic review is registered with PROSPERO [55] and follows the Preferred Reporting Items for Systematic Reviews and Meta-Analyses (PRISMA 2020 Statement) standards [56] and the MOOSE guidelines for meta-analysis of observational studies [57]. We have included the PRISMA 2020 Checklist in the Supplemental Materials for completeness (Table S1); however, not all items fit in the structure as this is a protocol rather than a completed review. Potentially relevant articles will be screened and selected by a trained medical epidemiologist (in-training clinical psychology doctoral student; HKS) who will search publicly available electronic databases, including MEDLINE via PubMed and OVID, EMBASE via Elsevier, Cumulative Index for Nursing and Allied Health Literature (CINAHL) via EBSCOhost, SCOPUS, PsychInfo, and the Cochrane Library. All of these are accessible through an institutional subscription at the Houston Academy of Medicine Texas Medical Center Library and available by federated login at UTHealth Houston.

### 2.1. Search Strategy

**Figure 1:**
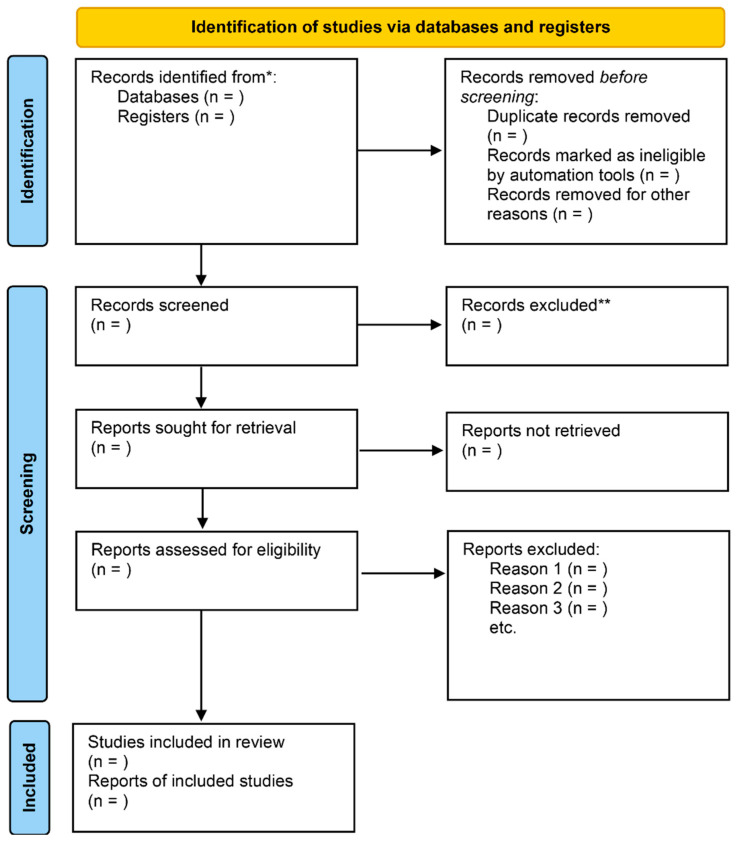
PRISMA 2020 flow diagram for systematic reviews. **Note**: Figure shows search results for this systematic review. (Adapted from Ref. [56]. This work is licensed under CC BY 4.0. To view a copy of this license, visit https://creativecommons.org/licenses/by/4.0/.).

Articles from the referenced databases will be searched from the establishment of each database to date. A search strategy will be developed by the research team, led by an experienced researcher who has familiarity with the methodology and conduct of systematic review and searching health science databases. The review team will work together to construct relevant vocabulary for the key concepts: (a) trauma- and stressor-related disorders and/or PTSD, etc.; and (b) ACEs (as defined above). A preliminary search might include Medical Subject Headings, such as (Cardiac Arrest OR Acute Cardiovascular Disease OR Coronary Disease OR Coronary Artery Disease OR Myocardial Ischemia OR Acute Coronary Syndrome OR Myocardial Infarction OR Aortic Dissection OR Acute Aortic Syndrome) AND (Stress Disorders, Post-Traumatic OR PTSD or Psychological stress) and their respective synonyms. Additionally, using a “snowball” technique [58], a manual bibliographic search of references from the included publications will be performed to identify other potentially relevant articles. Only studies published in English, or with an abstract in English, with ascertainable relevant information, will be eligible for inclusion. Figure 1 demonstrates a pictorial representation of the search and study methodology that will be utilized in conducting this review under the PRISMA guidelines.

### 2.2. Population and Study Selection

Studies will be eligible for inclusion if they describe PTSD (and/or trauma- and stressor-related disorders) as a consequence of any of the ACE component events. Eligible study designs would include both randomized controlled trials and observational studies (case–control, cross-sectional studies, case series, case reports, prospective cohort studies).

Furthermore, for Specific Aim 2, all articles that present results of management of PTSD and/or trauma- and stressor-related disorders developed as a consequence of an ACE will be included. All above-mentioned study designs will be eligible provided they measure treatment of PTSD or stress symptoms due to an ACE and estimate its association with mental health and/or CVD outcomes.

### 2.3. Inclusion Criteria

1. Studies conducted on adult participants >/= 18 years suffering from PTSD or stress disorders (acute stress disorder, adjustment disorder, or PTSS) after surviving a life-threatening acute cardiovascular event of any type. The spectrum of these will include, as previously described, CA, acute MI (STEMI or NSTEMI), UA, AAS, acute aortic dissection, penetrating aortic ulcer, acute intramural hematoma. These will include studies with the co-occurrence of diagnosis of stress-related disorders and an acute cardiovascular event.
2. Peer-reviewed empirical journal articles with either experimental or observational study designs, as described above, will be included except for reviews and meta-analyses. We will review the relevant literature summary articles to identify includable studies from their bibliographies.
3. Articles that present data on the prevalence of incident PTSD as a consequence of ACEs.
4. Studies with the full text published in the English language will be included. Additionally, studies with an abstract in English, with ascertainable relevant information, will be included.
5. No restrictions on publication period will be applied.
6. Only for Specific Aim 2—articles that include intervention that targets management of PTSD or stress-related disorder.
7. Only for Specific Aim 2—articles that include at least one post-treatment outcome (mental health symptoms or outcomes or CVD-specific outcomes).

### 2.4. Exclusion Criteria

1. Articles with the full text in a non-English language with required information not ascertainable from abstract or tables.
2. Articles that do not present original research, and systematic reviews or similar article types.
3. Participants under 18 years of age if adults are not reported separately.
4. Patients presented with pre-existing PTSD and not incident PTSD as a consequence of ACEs.
5. Articles that are primarily physiological or animal studies.
6. Only for Specific Aim 2—articles not reporting results of psychological intervention focused on the management of PTSD symptoms.

### 2.5. Analytical and Data Extraction Methods

To address each of the above-mentioned specific aims, data will be extracted, reviewed, assessed, analyzed and reported. The following is a plan for each step of these methods.

### 2.6. Analysis Plan: Specific Aim 1

Using all of the articles found and included based on the above-mentioned search strategy and eligibility criteria, the review will summarize the existing literature on the occurrence and characterization or presentation of ACEI-PTSD, including CA, ACS, and AAS. Pooled estimates of prevalence will only be computed from studies with appropriate denominators for these estimates. This will be completed using table format and text. Analysis will include tabulation and reporting of descriptive statistics regarding the prevalence of ACEI-PTSD and other outcomes described above. Study-weighting for summary estimates will be computed using SAS or R software. Any groupwise outcomes reported would be summarized using forest plots and evaluated for heterogeneity using I^2^.

### 2.7. Analysis Plan: Specific Aim 2(a)

In the first part of Study Aim 2, using the subset of articles addressing interventions from all the articles found and included, the review will summarize the existing literature on treatments being used specifically for ACEI-PTSD and categorize them (e.g., trauma-focused vs. non-trauma-focused EBI vs. non-EBI) and assess their effectiveness for PTSD symptom reduction. Study weighting for summary estimates will be computed using SAS or R software. Any groupwise outcomes reported would be summarized using forest plots and evaluated for heterogeneity using I^2^.

For Aim 2, this review will only consider studies that evaluate outcomes of any psychosocial (pharmacological and/or psychological) interventions or combination of interventions focusing on the management of incident stress-related disorders or on PTSD occurring because of an ACE. Included interventions might be different in type (trauma-focused vs. not, evidence based intervention vs. not, ranging from cognitive behavioral therapies to third-wave CBT techniques, e.g., acceptance and commitment therapy or mindfulness-based stress reduction), duration, delivery timing (days/months after acute event), setting (inpatient, outpatient, during rehabilitation, etc.), format (individual vs. group), mode (in person vs. telehealth vs. self-paced web based, hybrid, etc.), and type of provider (licensed clinical psychologists, credentialed counselors, health workers with some training, trainees, etc.). However, all will be considered—provided all other inclusion criteria listed above are met. In a more mature field, it would be useful to exclude vague intervention descriptions, such as therapeutic counseling or educational interventions, and other more general health-promotion interventions, such as unstructured exercise counseling and smoking cessation counseling. However, in this developing subject matter area, it seems reasonable to include them for narrative review purposes to capture what practitioners are reporting from the field. Health promotion efforts, or interventions that are clearly protocolized and structured, will be includable in the analytical part of the review.

### 2.8. Analysis Plan: Specific Aim 2(b)

In the second step of Study Aim 2, if the extracted data are sufficient to support the meta-analysis on treatment effectiveness, SAS or R software will be used to perform the computations. Random-effects modeling with the generation of forest plots and weights for studies according to sample size/numbers of events contributed will be used to aggregate the per-study data. Heterogeneity among studies, and its contribution to overall observed variance, will be quantified using the I^2^ statistic. Heterogeneity will be classified as high when I^2^ > 75%, moderate for I^2^ 50–74% and low if I^2^ < 50%. If data are inadequate to support the meta-analysis for key outcomes, explanatory tables and narrative reporting will be developed. Sensitivity analyses will be performed as appropriate to assess the influence of individual studies on the conclusions.

We anticipate that most studies—especially comparative or interventional studies—will be small and will consequently have unstable estimates. We will use the Freeman–Tukey double arcsine transformation to stabilize estimates to avoid the disproportionate influence of extreme values on pooled estimates [59]. Across all aims, effect estimates for dichotomous outcomes will be converted to odds ratios using standard formulae, and continuous data will be converted to standardized mean differences with 95% confidence intervals [60]. In addition to quantitative heterogeneity analyses (I^2^), we will assess heterogeneity qualitatively by reviewing study populations, treatment protocols and the directness of measurability (clearly defined vs. vague criteria) of outcome measures.

### 2.9. Analysis Plan: Specific Aim 3

Results of the analyses described for Aim 2, 2(a) and 2(b) will be reviewed for consistency and relevance to specific outcomes. For example, if CBT EBIs are identified as being effective for reducing PTSD symptoms, such as intrusions or avoidance, improving medication adherence and reducing re-hospitalization, then tables will be constructed that show each of these categories of effect and their relevant strength of association linking the intervention with that outcome. In addition, level of evidence quality using the GRADE criteria described below will be used to annotate the strength of any recommendations provided. Where data are unavailable or inadequate to provide guidance for practice, this will be further annotated, and recommendations for future research will be presented.

### 2.10. Data Extraction and Management

Two investigators will independently review all titles and/or abstracts of the retrieved studies using the aforementioned search strategy and full text retrieval of all potentially included studies will be performed and independently reviewed for assessment of eligibility for final analysis and assessment of study quality and level of evidence. Disagreements over a study’s eligibility will be resolved by a third reviewer and full consensus will be reached before proceeding to the next stage of review. Articles that clearly do not meet the inclusion criteria will be excluded and the number of excluded articles at each level of review (at the title and abstract level, full text reviewed, etc.) will be tracked and presented following the PRISMA guidelines. (Figure 1).

Extracted datapoints will include: author information; journal; publication year; study design; patient demographics; number of patients (overall and in intervention and control arms); clinical profile (clinical presentation, mental health symptoms, ACEs and trauma-/stressor-related specific diagnosis, etc.); location and setting; study methods; intervention details (type, format, setting, delivery, who performed, duration, frequency); screening and diagnostic tools (for mental health diagnosis and assessment); outcome measure tools; and critical outcome frequencies and measures. All extracted data will be independently verified by both reviewers and inconsistencies will be resolved through discussion and/or arbitration by a third reviewer. All attempts will be made to contact corresponding authors of original publications to request additional or missing information in the event this is necessary for a study to meet inclusion criteria.

### 2.11. Quality Assessment

All selected studies will be critically and independently assessed for risk of bias and quality of evidence using GRADE criteria for all quantified outcomes—assessment of bias, publication bias, inconsistency, imprecision, and indirectness [61]. The recommendations developed by the GRADE working group aim to rate the “certainty of evidence” presented for specific outcomes—and these criteria are frequently used to support practice guidelines [62]. GRADE specifies 4 levels of study quality—high, moderate, low and very low—and is primarily used to judge systematic reviews that evaluate effectiveness of interventions. The rating process is primarily assigned a level based on the rigor of the study design (for instance, RCTs will start as “high” and observational designs will start as “low”) but can be downgraded or upgraded, depending on specific factors, such as large effect size, confounding, risk of bias, indirectness, publication bias, etc. For bias assessment of individual studies, we will use Cochrane RoB2 for RCTs, ROBINS-I for nonrandomized comparative interventional studies when applicable [63,64]. Publication bias will be assessed using funnel plots and Egger’s test. Regardless of the methodological quality of the included studies, all studies will undergo data extraction and synthesis to the extent possible.

## 3. Discussion

PTSD and CVD are common and create a dangerous synergy in which each worsens the other, leading to a multiplier effect, particularly in cases in which PTSD arises as a consequence of ACE. Both are major public health burdens but their combined occurrence can significantly escalate morbidity and mortality, which demands that an integrated care approach be developed. Despite the importance of these interacting phenomena, the literature on these topics is fragmented and gaps remain in the understanding of the link between PTSD and ACEs.

Important gaps in understanding the interplay between these problems include a lack of aortic syndromes in current general definitions of ACSs, poor dissemination and use of the EST model in the literature and, consequently, a lack of empirical evidence on the prevalence and differences in presentation of medical trauma-induced PTSD. Although trauma-focused interventions have been shown to be effective for managing PTSD symptoms generally, evidence regarding the applicability and effectiveness of trauma-focused approaches to managing ACEI-PTSD is lacking. All of these gaps can be addressed in a systematic review.

The science surrounding PTSD as a consequence of medical trauma (ACEs in particular) is in its infancy and is rapidly evolving. The literature is fragmented and appears focused on a small group of clinical problems. A comprehensive systematic review is required to identify, organize and evaluate the literature and—if of sufficient quality and quantitative completeness—to help assess current treatment strategies and identify areas for the dissemination of findings and future research.

We are publishing this protocol to both call attention to the need for a comprehensive literature summary to inform practice and to prespecify our search plan to serve as a procedural map. Any revisions to the plan during review construction will be documented with the results of the review. Finally, we will identify the current state-of-the-art treatments, which have been subjected to rigorous study, and propose strategies for future work in areas of uncertainty.

## Data Availability

The original contributions presented in this study are included in the article/Supplementary Materials. Further inquiries can be directed to the corresponding author.

## Supplementary Materials

The following supporting information can be downloaded at https://www.mdpi.com/article/10.3390/jcm15082962/s1: Table S1: PRISMA 2020 Checklist.

## Abbreviations

AAS	acute aortic syndrome
ACE	acute cardiovascular event
AD	acute dissection
CA	cardiac arrest
CBT	cognitive behavioral therapy
CVD	cardiovascular disease
ECG	electrocardiogram
EFT	emotionally focused therapy
EST	Enduring Somatic Threat
ICD	implantable cardioverter defibrillator
IMH	intramural hematoma
MI	myocardial infarction
PAR	population attributable risk
PAU	penetrating aortic ulcer
PTSD	post-traumatic stress disorder
UA	unstable angina
